# ‘A disease like any other’ traditional, complementary and alternative medicine use and perspectives in the context of COVID-19 among the Congolese community in Belgium

**DOI:** 10.1186/s13002-022-00530-y

**Published:** 2022-04-07

**Authors:** Emiel De Meyer, Patrick Van Damme, Eduardo de la Peña, Melissa Ceuterick

**Affiliations:** 1 Department of Plants and Crops, Ghent University, 9000 Ghent, Belgium; 2 Faculty of Tropical AgriSciences (FTA), Czech University of Life Sciences Prague, 165 00 Prague, Czech Republic; 3 Institute for Subtropical and Mediterranean Horticulture, IHSM-UMA-CSIC, Finca Experimental La Mayora, 29750 Algarrobo-Costa, Malaga Spain; 4 Department of Sociology, Health and Demographic Research, Ghent University, Sint-Pietersnieuwstraat 41, 9000 Ghent, Belgium

**Keywords:** Urban ethnobotany, Congolese community, Belgium, COVID-19

## Abstract

**Background:**

As a hard-hit area during the COVID-19 pandemic, Belgium knew the highest mortality among people from sub-Saharan African descent, compared to any other group living in the country. After migration, people often maintain traditional perceptions and habits regarding health and healthcare, resulting in a high prevalence of traditional, complementary and alternative medicine use among different migrant communities in northern urban settings. Despite being the largest community of sub-Saharan African descent in Belgium, little is known on ethnobotanical practices of the Belgian Congolese community. We therefore conducted an exploratory study on the use of medicinal plants in the context of COVID-19 and perceptions on this new disease among members of the Congolese community in Belgium.

**Methods:**

We conducted 16 in-depth semi-structured interviews with people of Congolese descent currently living in Belgium. Participants were selected using purposive sampling. Medicinal plant use in the context of COVID-19 was recorded through free-listing. Data on narratives, ideas and perceptions on the origin, cause/aetiology and overall measures against COVID-19 (including vaccination) were collected. Interview transcripts were analysed using thematic analysis.

**Results:**

Four overarching themes emerged from our data. Firstly, participants perceived the representation of the severity of COVID-19 by the Belgian media and government—and by extend by all governmental agencies in the global north—as exaggerated. As a result, traditional and complementary treatments were seen as feasible options to treat symptoms of the disease. Fifteen forms of traditional, complementary and alternative medicine were documented, of which thirteen were plants. Participants seem to fold back on their Congolese identity and traditional knowledge in seeking coping strategies to deal with the COVID-19 pandemic. Finally, institutional postcolonial distrust did not only seem to lead to distrust in official messages on the COVID-19 pandemic but also to feelings of vaccination hesitancy.

**Conclusion:**

In the context of the COVID-19 pandemic, participants in our study retreated to, reshaped and adapted traditional and culture-bound knowledge. This study suggests that the fragile and sensitive relationship between sub-Saharan African migrant groups and other social/ethnic groups in Belgium might play a role in their sensitivity to health-threatening situations, such as the COVID-19 pandemic.

## Introduction

During the COVID-19 pandemic, Belgium has been a hard-hit area, with 3.6 million confirmed cases and 30,200 deaths up to the time of writing this article (March 2021) [1]. During the first infection wave, in spring 2020, Vanthomme et al. [2] reported a significantly higher age-standardized mortality rate (ASMR) among both men and women of Sub-Sahara African communities than any other group living in Belgium. They state that the excess mortality during the first COVID-19 infection wave among migrant communities in Belgium resulted from a complex interplay between multiple factors. They argue that socio-economic and socio-demographic elements, together with cultural behaviour, access to health services, precarious job and housing conditions, travelling and the wider urban context, led to a higher vulnerability of these communities to get infected with and recover from COVID-19 [2]. Woodhead et al. [3] reported COVID-19 vaccination hesitancy among health care workers in the UK from racial and ethnic minority groups and argued that this risks aggravating health and social inequalities.

Most cities in the global north, and by extent urban settings, have become super-diverse places where complex multicultural realities have emerged and many political, social and religious traditions are mixed [4, 5]. Weckmuller et al. [6] state that globalization and urbanization, resulting in increased accessibility to biomedical health care and improved transportation and connectivity, may result in a loss of non-biomedical medicinal knowledge. However, cultural concepts and habits on health, illness and medicine, including health care-seeking strategies, are usually preserved or rethought after migration [7–9]. Traditional and complementary medicine (TCAM) in developing countries consists mainly of plants and plant-derived products, and as such medicinal plant use among diverse migrant communities prevails in many Western urban settings [10–14]. There are several possible reasons explaining medicinal plant use by communities with a migratory background. This continued use has been explained as an identity-seeking strategy [15], as a way to treat culturally bound syndromes, or because of the (believed) effective pharmacological effect of known medicinal plants and their derived products [14]. Besides searching for maintaining cultural continuity, migrant communities also seem to adopt formerly unknown uses of TCAM by assimilating new species from their present living environments, and replacing others, whereby both accessibility to plants and cultural forces seem to play a role in the adaptation mechanism [16]. It has been shown that medical pluralism of different ethnic groups in a super-diverse environment leads to complex and contingent health care-seeking pathways [17–19]. Furthermore, Vandebroek et al. [20] reported an increased use of medicinal plants during the COVID-19 pandemic, especially in hard-hit areas. A main driver behind this increase was the rapid and wide dissemination of information about home remedies through social media, YouTube, TV and word of mouth [21].

As a result of a long colonial history [22], the Congolese (Democratic Republic of the Congo, hereafter mentioned as DR Congo) community is the largest community of sub-Saharan African descent in Belgium, with an estimated 87,440 people living in Belgium in 2020 (Figures from the National Register, drawn up by Statbel, edited and calculated by Myria) (personal communication, 2021). Primary health care in DR Congo consists mainly of TCAM, of which at least a part is preserved after migration [14, 23, 24]. Van Andel and Fundiko [14] report a high prevalence of medicinal plant use among the Congolese community in Belgium, in which Matongé, the Congolese neighbourhood in Brussels (named after the vibrant eponymous quarter in Kinshasa, DR Congo), acts as a hub in the trade of Congolese medicinal plants. As TCAM use and health care-seeking strategies among this group are highly under-investigated subjects and since this community was so hard-hit by the pandemic, this paper aims to:

- document current medicinal plant knowledge and use among the Congolese community to prevent, cure, or treat symptoms of COVID-19.
- link health care practices to those known in the DR Congo.
- explore the overall perception of feasible health choices in the light of the pandemic within the Congolese community in Belgium.

## Materials and methods

### Interviews

Data on medicinal plant use and perceptions on COVID-19 of the Congolese community in Belgium were collected during fieldwork that took place between September 2020 and April 2021. Eligibility criteria included: a minimum age of 18 years, having at least one parent from Congolese (DR Congo) descent, permanently residing in Belgium, and having used herbal medicine in Belgium. We designed a purposive sample to obtain maximum variation in gender, age, professional background and place of residence [25]. The first participants were searched by approaching individuals and non-profit organizations (NPO) and recruited through subsequent snowball sampling [26]. Such a personal network-based approach for recruitment was deemed necessary, as participants were hard-to-reach due to governmental restrictions in the context of the pandemic [26]. Moreover, to avoid sampling within a closed personal network [27] and to obtain a theoretically representative sample of the Congolese community, the snowball sampling departed from different key participants or starting points and we continued recruiting new profiles based on the theoretical sampling criteria that were still missing. Depending on the Belgian government measures against COVID-19 at the moment of interviewing, interviews either took place online or in-person. Online interviews were conducted using videotelephony software. Before the start of every interview, informed consent was obtained verbally. After the interviews, a signed informed consent document was obtained. For the interviews performed virtually, a standardized informed consent document was sent in advance, whereas a scan of the signed version was sent after the interview by every participant.

All interviews were audio-recorded after obtaining written informed consent. Online interviews were digitally recorded. During the interviews, meta-data/observations were noted manually. All interviews were fully transcribed and anonymized.

Information was sought about the use of herbal remedies to prevent, cure and fight symptoms of COVID-19. Participants were asked to free-list medicinal plants they knew or had already used against COVID-19. They were asked to describe the preparation, administration and medical use (preventive/curative/fight symptoms) of the different herbal remedies. They were also asked if they had had COVID-19. In addition, participants’ ideas and perceptions on the origin and cause of COVID-19 as well as on the overall situation and measures against COVID-19 were collected. Finally, they were asked about their personal ideas on vaccination. Interviews were conducted until no new qualitative data were obtained by conducting more interviews, and qualitative data saturation was reached (i.e. when a theme reoccurred twice).

### Data analysis

Interview transcripts were analysed in the language in which they were conducted. Qualitative data were analysed using thematic analysis [28]. Given the exploratory nature of this study, we deemed this approach particularly relevant to identify prevailing ideas and practices. Transcripts were coded in different phases as described by Clarke and Braun [28]. Draft themes were discussed with all authors in an iterative process and led to four final themes which are elaborated in the Results section. Relevant parts of the transcriptions were translated into English for use as quotes.

### Plant identification

A voucher collection was assembled of all plant species that were named during the interviews and that were available in Belgium. Physical plant samples were collected in African shops in Matongé, or offered by the participants. All collected physical samples were identified using PROTA4U [29] and Flore d’Afrique Centrale [30] and were included in both a physical and digital voucher collection (see Table 1). Of plants that were mentioned by participants but that were not physically (or abundantly) available in Belgium at the time of the fieldwork, clearly identified pictures of the plant habitus and plant parts were sought online together with the participants to determine the plant species through visual identification. Of the two non-available plant species, vernacular names were linked to scientific botanical names based on the knowledge of the authors, confirmed by the scientific ethnobotanical literature.

**Table 1:** Demographics of study participants

Factors	
Total participants	16
Female	8
Male	8
Average age	48 (SD: 14.54)
Age range (years)	28–70
First generation in Belgium	13
Time of residence: range (years)	8–50 (average: 23)
Second generation in Belgium	3

### Literature review

The identified species were then compared to the ethnobotanical literature on the Congolese pharmacopoeia. An online literature search was conducted using ISI Web of Science, JSTOR and PubMed for scientific articles (keywords: Congolese, ethnobotany, medicinal plants). Vulgarized works were not included, as there is no certainty about the correctness of the plant identification. General scientific works on medicinal plants from DR Congo were included, as well as works on medicinal plant use in specific regions. Scientific works confirming the use of a plant species are included in Table 1.

## Results

### Interviews

In total, 16 qualitative in-depth, semi-structured interviews were conducted using open-ended questions with people from Congolese descent. Eight interviews were performed online, while eight in-person. Interviews lasted between 30 min and 2 hours and were conducted in French or Dutch, depending on participants’ preferences. Table 1 shows that three participants were born in Belgium (second generation in Belgium). For the other thirteen participants, their residence time in Belgium was between 8 and 50 years. An equal amount of women and men were interviewed (8:8). The age of participants ranged between 27 and 70 years (average 48 years).

Eight participants held a master degree, four of them in Belgium and four in the DR Congo. Two participants held a bachelor degree (obtained in the DR Congo). Six participants had a secondary education diploma. At the time of the interviews, four participants were retired, and two were unemployed. One participant had a job in the secondary sector. The other participants had various jobs in the tertiary sector; two of them were general practitioners. The participants lived in and around Antwerp, Brussels, Ghent and Ostend.

Our analysis led to four overarching themes: (1) reinterpretation of TCAM in the light of COVID-19; (2) information seeking through transnational and digital networks; (3) ‘A disease like any other’: Corona from/in a Congolese perspective’; and (4) vaccination hesitancy.

1. Reinterpretation of TCAM in the light of COVID-19

None of our participants had tested positive for COVID-19 at the time of the interview, but all knew people in their social network who had suffered from COVID-19. A majority of participants saw TCAM as the only option for adequate health care in the context of COVID-19, and as the better and healthier option compared to getting vaccinated. Likewise, the majority (13 out of 16) of the interviewees mentioned TCAM to prevent, cure or relieve symptoms of COVID-19 (or a combination of the previous actions). A total of 15 different forms of TCAM were mentioned in relation to COVID-19, of which thirteen were plants. All TCAM are listed with their family, scientific and vernacular name, times mentioned, whether they are used in Belgium, effect against COVID-19, link with malaria, origin of the plants and whether their use is already described in African/Congolese pharmacopeia in Table 1. We recorded thirteen different plant species, of which two could only be identified up to genus level, because multiple species within the genera *Eucalyptus* and *Citrus* were used. The most frequently mentioned plant species were *Artemisia annua* L. (*n* = 7), *Citrus* spp. (*n* = 5) and *Zingiber officinale* Roscoe (*n* = 5). Plants were mainly obtained through supermarkets (ginger, *Citrus* spp.), African shops or informal networks. The only TCAM that we did not record as a plant was ‘confo®’, a mix of different (mentholated) essential oils sold online and used as an insect repellent, and Vicks®. The latter is an American brand of over-the-counter medicines. Of two forms of TCAM, only the use in DR Congo was mentioned. These plants were used curatively against COVID-19. All but these two plant species were physically available in Belgium. The participants obtained the various TCAM by (1) bringing them from DR Congo themselves, (2) informal networking, and (3) buying them in African shops (in Matongé, Brussels). The two non-available plant species (*Tetradenia riparia* (Hochst.) Codd. and *Psidium guajava* L. (leaves)) were identified through visual identification.

The origin and geographic distribution of the documented species are shown in Table 1*.* Four out of thirteen plant species used in the context of COVID-19 are native to the DR Congo (i.e. *Picralima nitida* T. Durand & H. Durand, *Morinda morindoides* (Baker) Milne-Redh., *Tetradenia riparia* (Hochst.) Codd. and *Ocimum gratissimum* L.); seven out of thirteen plant species are not native to Africa (and the DR Congo), but have a pantropical (*Moringa oleifera* Lam., *Eucalyptus* spp., *Psidium guajava* L., *Citrus* spp., *Zingiber officinale* Roscoe) or global distribution (*Allium cepa* L. and *Allium sativum* L.). The other species (*Allium schoenoprasum* L. and *Artemisia annua* L.) have no well-founded geographic distribution in Africa. Furthermore, twelve of the thirteen plants found in this study are also described for different purposes (others than COVID-19) in the Congolese ethnobotanical literature and pharmacopeia (see Table 1). The only plant whose use has not been described was *Allium schoenoprasum* L.

The choice for these specific remedies can be explained by the participants’ view on the aetiology and clinical picture of COVID. Several participants considered COVID-19 and malaria to be similar diseases that cause the same symptoms and thus should be treated similarly. Many participants compared the symptoms of COVID-19 with the (combined) symptoms of malaria and flu.

‘Corona, flu, and malaria all cause the same symptoms. When you have malaria, you don't have taste, you have a high fever, and if you have both malaria and the flu, you are really like someone who has corona, because malaria associated with the flu… you have no taste and you have a high fever, you have trouble breathing… all that is said about corona symptoms ... And yet there are people who cure what they say about corona symptoms.’ – woman, 45, 1^st^ generation in Belgium

Many forms of TCAM (*n* = 5) were linked to COVID-19 because of their supposed effect against malaria. Particularly, *Artemisia annua* L. was mentioned seven times to work both preventively and curatively against COVID-19, derived from the fact that this plant or plant-derived products are frequently used against malaria (Table 2).

‘Artemisia is used against malaria, but we have discovered that it also works against corona.’ – woman, 70, 1^st^ generation in Belgium

‘…like my aunt, she had corona, she had it twice and she treated it as if she had had malaria.’ – woman, 36, 1^st^ generation in Belgium

**Table 2:** List of TCAM used against COVID-19 mentioned by the Congolese community in Belgium

No.	Plant family	*Scientific name*	Recorded vernacular name	Origin and geographic distribution	Frequency of quotation	Mode of preparation	Used in Belgium?	Curative/preventive/ against symptoms	Link with malaria?	Use documented in Congolese pharmacopoeia(s)	Voucher collection reference number *Annex 1*
1	Alliaceae	*Allium cepa* L	Oignon	Gl dst	X	Di	Yes	Preventive	No	[23, 24]	Vc1
2		*Allium sativum* L	Ail	Gl dst	X	Di	Yes	Preventive	No	[23, 24]	Vc2
3		*Allium schoenoprasum* L	Ciboulette/Bieslook	Am, As, Eu	X	Di	Yes	Preventive	No	Not documented, new use	Vc3
4	Apocynaceae	*Picralima nitida (Stapf)* T.Durand & H.Durand	Bonobo	Af (DRC)	X	Di	Yes	Curative	Yes	[30, 31]	Vc4
5	Asteraceae	*Artemisia annua* L	Artemisia (fr.)	Eu, As	XXXX	Dc	Yes	Curative/preventive	Yes	[23, 30]	Vc5
6	Lamiaceae	*Ocimum* gratissimum L	Lumba lumba	Af, As	X	Di, Dc	Yes	Curative/ against symptoms	No	[24, 30, 31]	Vc6
7		*Tetradenia riparia* (Hochst.) Codd	Mutuzo	Af (DRC)	X	Di	No	Curative	Yes	[30, 32]	N.A
8	Moringaceae	*Moringa oleifera* Lam	Moringa	As, Pantr dst	X	Di, Dc	Yes	Preventive	No	[33, 34]	Vc7
9	Myrtaceae	*Eucalyptus* spp.	Eucalyptus	Au, Pantr dst	X	Sb	Yes	Curative/against symptoms	No	[30]	Vc8
10		*Psidium guajava* L	Feuilles de goyave	Am, Pantr dst	X	Sb	No	Curative	Yes	[30, 32]	N.A
11	Rubiaceae	*Morinda morindoides* (Baker) Milne-Redh	Kongo bololo	Af (DRC)	X	Dc	Yes	Curative	Yes	[30, 31]	Vc9
12	Rutaceae	*Citrus* spp*.*	Citron	Pantr dst	XXX	Di, Dc	Yes	Curative/preventive	No	[23, 30–32]	Vc10
13	Zingiberaceae	*Zingiber officinale* Roscoe	Gember/gingembre/tangawisi	As, Pantr dst	XXX	Di, Dc	Yes	Preventive/ against symptoms	No	[23, 24]	Vc11
14	N.A	N.A	Confo® (essential oils)	N.A	XX	Sb	Yes	Curative/preventive/against symptoms	No	N.A	Vc12
15	N.A	N.A	Vicks®	N.A	X	Sb	Yes	Curative/preventive	No	N.A	Vc13

Origin and geographic distribution: Gl dst.: Global distribution; Eu: Europe; As: Asia; Au: Australia; Af: Africa; Am: America; Pantr dst: Pantropic distribution; X = use quoted by less than 15% of the informants; XX = use quoted by more than 15% and less than 30% of the informants; XXX: use quoted by more than 30% and less than 45% of the informants; XXXX: use quoted by more than 45% of the informants. Mode of preparation: Dc: decoction; Di: direct ingestion; Sb: steam bath

Likewise, four other plant species (*Morinda morindoides* (*Kongo bololo*), *Picralima nitida,* (*bonobo*)*, Tetradenia riparia* (*mutuzo*) and *Psidium guajava* (*feuilles de goyave*)), all documented for their use to treat malaria, were mentioned for their assumed capacity of healing COVID-19. Several participants also referred to plant-derived medication used to treat malaria when talking about plants used against COVID-19. One participant stated that people use anything that contains quinine, a molecule used to treat malaria. Another participant explained that *Kongo bololo* is a natural container of chloroquine, a biomedicine used to prevent and treat malaria. A different participant indicated that in DR Congo, less people die, because they are resistant from taking medicine (*Artemisia*) against malaria.

‘People use anything that contains quinine. ‘– woman, 43, 1^st^ generation in Belgium

‘Corona is like malaria. So, it is really the Kongo bololo that works a 100% in three days. Kongo bololo is chloroquine in the raw state ‘– man, 28, 2^nd^ generation in Belgium

Furthermore, participants put a lot of faith in these remedies, as evidenced from the following quote:

‘People don't die like here. And so, there is a man called Doctor Raoul who revolted ... He says that Africans, if they don’t die of COVID, it is because they have taken too many antimalarial drugs. Because there are molecules in there, formulas I can't explain, it's magic. So, I trust using Artemisia.’ - woman, 61, 1st generation in Belgium

(2) Information seeking through transnational and digital networks

Participants predominantly sought information on ways to deal with COVID-19, and by extension the pandemic and its consequences through informal networks within the Congolese community, including acquaintances living in Congo. Social (digital) networks played an important role in information dissemination about COVID-19 among people of Congolese descent. WhatsApp groups within the Congolese community and acquaintances in Congo were the main information dissemination mode within this group. Stories, pictures and movies were shared through these groups, causing their rapid spread beyond borders. Participants often mentioned anecdotal information from within the community, or in the DR Congo. Perceptions and ideas originated in the DR Congo were regularly adopted by participants in Belgium and stated as being ‘true’. Likewise, our participants received information on TCAM through these networks. Additionally, information on health and health care (e.g. the supposed link between malaria and COVID-19) was sought online. Participants’ motifs often started with *‘I heard…’* or *‘I think…’*. This shows that people’s own ‘truths’ were built based on information they received through informal and unofficial channels. One participant explained that she received messages through WhatsApp about *Artemisia* from Congolese people she didn’t know very well.

‘They contacted me (through WhatsApp) here to try to help buy the Artemisia. But since they were people who I didn't know very well, I refused’ – woman, 70, 1^st^ generation in Belgium

(3) ‘A disease like any other’: COVID-19 in a Congolese perspective

Many participants in this study viewed the way the COVID-19 pandemic was handled by the Belgian government as exaggerated and perceived COVID-19 as ‘any other’ or ‘yet another’ potentially mortal infectious disease. Participants compared COVID-19 with diseases like the flu, malaria, AIDS and Ebola, to put the fact that people recover or die from certain diseases into perspective. One participant explained the way the pandemic was handled in Europe by suggesting that in Europe, there are no other infectious diseases that cause so many deaths, as opposed to in DR Congo.

‘I think what Corona has done ... We have ... so enlarged a disease that could be a normal disease like any other. Because today we're talking about ... there are a lot of people dying from Corona, but there have been a lot of people who have died of the flu too. Because there are people who die of malaria too. But here you yourself do not know about malaria. Yet there are people who are recovering from malaria in the Congo. Not everyone dies. But, here, we really have a disease… Me, all I hear, effects of… symptoms of Corona, these are the symptoms of malaria plus the flu… together… and the flu can be treated and malaria can be treated. There are people who die from it, yes… But there are also people who are healed from it.’ – Woman, 45, 1^st^ generation in Belgium

‘In Congo, they are busy with other things. They know pandemics. They know Ebola, AIDS , all these things…’ – woman, 30, 2^nd^ generation in Belgium

One participant explained the way the pandemic is handled in Belgium, by comparing the difference in perceptions of death in Congo and Belgium. This participant clarified that living circumstances in Europe have become so good and people live so long that it has almost become abnormal that people die. While death in Africa is seen as being ‘part of everyday life’, it has become taboo in Europe, as illustrated in this quote:

‘We are still human beings who at some point have to stop. The mentality in Europe is, in fact, that we always try to live longer, and in better living conditions. Okay, but death is not part of life in Europe, instead it’s very much taboo. Ultimately, when you listen to the news, in the twenty-first century in Europe, there are no longer people who have to die. While in Africa, when a week has passed without losing one of your acquaintances, it’s been an exceptional week. Death… death is in fact present in everyday life in Africa. Here it is almost abnormal.’ – woman, 56, 1^st^ generation in Belgium

Participants often started explaining their point of view with: *‘The African community says…’* or *‘We, Africans think…’* or *‘The mamas say…’*, which further reveals an ‘us versus them dichotomy’. ‘Us’ being the black community, and ‘them’ being the white community. Two participants explained this ‘us versus them’ dichotomy extensively, as illustrated in the quotes below. They suggested that European governments idly claim to possess the right knowledge to treat COVID-19, but that there are solutions that could come from outside this European domain of knowledge. They state that *‘Europe can learn from Africa’*, and in particular from traditional knowledge on TCAM, as outlined in the following quotes:

‘Yes, but very often it’s a bit complicated between the two cultures. Black and white, it always is complicated. Usually, white people think they know everything, so he (ed. a man she is talking about) knows: normally he is against everything. He cannot learn anything from an African. But I find it really doubtful. Because today for example with Corona, people in Africa… There is Corona, but people are not as sick as here in Europe. This is to say that with everything black people also know. It helps a lot and it heals a lot. So, the European countries have a lot to learn, also from Africa. On medicine, too. Because there are things that heal there. There's so much sickness out there that people, they just don't feel right and… are near life and death healing. But when they come here, they die. I know of two cases. There is a man who is Belgian, therefore a white, a Belgian who lives in the Congo. And there is another man who is black, Congolese, both, they were in Africa. They both fell ill. The white man stayed behind to seek treatment in the Congo. And the black man came back home to England for treatment. It was the one in England who died, hey. It was he who was in the Congo, who was healed. He is not dead. He is well and in good health.’ - woman, 45, 1^st^ generation in Belgium

‘The mamas said to me: ‘We know plants that they don’t know.’ There are a lot of plants that we could use and improve, I believe. I think Europe can learn from Africa. The mamas know everything, but they don’t know the composition of all the plants.’ – woman, 61, 1^st^ generation in Belgium

One participant also criticized how he feels the Congolese community is discriminated and marginalized in Belgium, saying that it is only now the first time a Belgian governmental message was translated in Lingala, one of the four national languages in DR Congo and spoken in Kinshasa. He stated that the Belgian government never *‘needed’* the Congolese in Belgium, but that now they need them to stop the virus spreading, they take into account the Congolese community.

‘It is the first time a governmental message was translated in Lingala (on the COVID-19 measures). That was never done before. They never needed us... but now they need to stop the spread of the virus because it harms them, they need us.‘ – man, 28, 1^st^ generation

(4) Vaccination hesitancy

Linked to the reliance on TCAM, the perceived relatively mild course of the disease and a distrust in governmental institutions, we observed different attitudes in the willingness of participants to get vaccinated. Most participants showed different levels of scepticism towards the development of the vaccine, and some even showed fear towards getting vaccinated. At the time of the interview, only two participants (who were general practitioners) said they would take the vaccine against corona. Some participants mentioned getting a vaccine was not necessary for them, because they had other ways of treating corona, or they believed they were immune because of their ethnicity.

‘I know how to treat corona in a natural way, so why would I take the vaccine?’ – man, 28, 2^nd^ generation in Belgium

‘Up to now, I haven’t been infected with corona. So, if I haven’t been infected up to now, should I take the vaccine?’ – man, 28, 1^st^ generation in Belgium

Most participants showed a lack of trust (and mentioned this is prevalent within the Congolese community) towards both the Belgian (and by extent all Western) and Congolese governments. Some participants doubted the governmental motives behind developing a vaccine and vaccinating people. They suggested that the reason for the vaccine’s quick development is *‘making money’*, not guaranteeing its safety, and that only if their freedom is curtailed when they refuse the vaccine, they will take it.

‘That's really not just ... a vaccine. We don't think it's an ordinary vaccine ... There is a little problem about that. It's really just about collecting money… That's it.’ – man, 55, 1^st^ generation in Belgium

‘If we have no other option and if it will limit our freedom, or if our children can no longer go to school, unless they have a vaccination… so if we are really limited, I will take it. ‘ – woman, 45, 1^st^ generation in Belgium

‘Personally, I’m afraid of it. I will only accept it after a few years.’ – woman, 61, 1^st^ generation in Belgium

One participant mentioned that there is a specific mistrust of Congolese, and African people in general, towards Western governmental practices. One participant claimed that Congolese people are afraid they would receive a different vaccine from European people. Another participant stated that the African (Congolese) people are convinced the vaccine is being used as a weapon to kill certain non-European people. A different participant said that in Congo, people are forced by police and military services to get the vaccine. The spread of hesitancy is reinforced by the use of social and other digital media. For example, a YouTube video that was sent by one of the participants after the interview and that was spread through WhatsApp within the Congolese community shows a panic outbreak in an elementary school as rumour had it that all children would get the vaccine at school. All this shows that Congolese people suspect European (and even Congolese) governmental practices regarding the vaccination will (try to) do harm to the Congolese. It is clear that stories from events happening in DR Congo reach the Congolese community in Belgium via social networks and that the emotions these stories evoke prevail beyond borders. This also illustrates a low institutional trust among the Congolese community in Belgium.

‘If people would see with their own eyes that white people get the same vaccine as black people, I think a lot more Congolese people would accept being vaccinated.’ – man, 28, 1^st^ generation in Belgium

‘The Africans think that ... they (i.e. the governments) want to get rid of certain people. They want to eliminate certain people… the categories of the people, the old people… or non-Europeans. They are going to be eliminated by a vaccine. This is what Africans say.' – man, 55, 1^st^ generation in Congo

‘Here in Congo there are military and police going to someone's house to give the vaccine, so… they reinforce…’ – woman, 54, 1^st^ generation in Belgium

Some participants on the other hand were relatively open about their willingness to get vaccinated. However, they were also sceptic about the quick development process of vaccines. Most participants had a wait-and-see attitude towards getting vaccinated. They mentioned that they would like to wait before getting vaccinated until they have the feeling the vaccine is safe.

‘It’s good that the vaccine is there and OK, we will see if it is effective, and then, why not?! We will see how it goes as time goes by. We will see.’ – woman, 43, 1^st^ generation in Belgium

Another critique toward the quick development of the vaccine, came from participants who viewed the reason why a vaccine is developed in such a short time as the fact that COVID-19 is a global problem. While the development of a medicine seemed to be non-urgent for diseases like Ebola that only hit the South.

‘Because I think it’s strange that in such a short time suddenly a panacea is created. While there are many pandemics... Ebola cannot be solved with a vaccine, so I think: 'what are the priorities? Is this because it is a global problem and that Ebola was more focused on the South? I just think it's all weird.’ - woman, 30, 2^nd^ generation in Belgium

## Discussion

Participants put the gravity of the coronavirus pandemic into perspective when comparing to other diseases that are highly prevalent in DR Congo. DR Congo has had multiple Ebola, polio, cholera and yellow fever outbreaks during the past 10 years [35]. Also, malaria is one of the main death causes (mainly among children < 5 years) with an estimated 631,000 deaths in Sub-Sahara Africa in 2015 [36]. Besides the link between COVID-19 and malaria, people stated that they have known diseases like Ebola and malaria and that, in respect to these diseases, the gravity of COVID-19 is exaggerated.

We discovered a strong ‘us-versus-them dichotomy’ in the narratives of the participants in our study, between a strong belief in information provided by the Congolese—and by extent African—communities in Belgium, and a very low trust in information provided by European governmental instances (including biomedical advisory boards). Racism and racial discrimination towards sub-Saharan African migrant groups is widespread in post-colonial Europe and Western settings in general [37] and can be evidenced from multiple social and societal aspects of everyday life, like prejudice, discrimination, political opposition and violence [38, 39]. All this strengthens the association (and thus identification) of individuals with their own in-group [40, 41]. In the light of this, TCAM use by the Congolese community (in general, so not confined to COVID-19) can be seen as a form of social identification and a w ay to strengthen the Congolese cultural identity [14]. In this context, the reinforcing effect of TCAM use on social identification can also be seen as an indirect coping strategy to maintain mental health and provide a sense of security [42]. Michels et al. [43] state that besides the concrete threat to people’s physical health, a reduced perceived situational control is one of the stressors people had to deal with during the COVID-19 pandemic. Medicinal plant use in this context can be addressed to the search for ways to reduce perceived lower situational control and can thus be seen as a culturally appropriate/culture-bound coping strategy.

In this light, participants in our study actively sought links between COVID-19 and their own cultural and traditional knowledge in search for strategies to deal with all aspects of COVID-19. All but one (*Allium schoenoprasum*) plants used against COVID-19 mentioned by participants were plants that are known and used medicinally in DR Congo [23, 31–34, 44]. The two non-plant TCAM, ‘*confo®’ and ‘Vicks*®’, were not (yet) documented as being used in the DR Congo. Due to their Eucalyptus-like properties, they make a logical option as a treatment as *Eucalyptus* spp. were used in steam baths to cure and fight symptoms of COVID-19. *Allium schoenoprasum* (chives) has no geographical distribution in Africa and was consequently not described in Congolese pharmacopeia. Its use can be explained due to its phytochemical analogies with *A. cepa* and *A. sativum*.

The link between the use of plants against malaria and their supposed use against COVID-19 seemingly emerges from the symptomatic similarities between the two diseases. Officially, similar symptoms are fever, fatigue, headache, and muscle and joint pain [45, 46], participants added dysgeusia and anosmia, which are officially reported as symptoms for COVID-19, but not for malaria [47]. This emic reinforcement of the link between COVID-19 and malaria can partly be attributed to the promoted use of *Artemisia annua* L., a plant that is well known and widely used for its antimalarial purposes [48] by multiple, mainly African, (governmental) authorities against COVID-19 (e.g. Burkina Faso [49]), Tanzania and Congo Brazzaville [50]. *A. annua* is not distributed throughout Africa in the wild [51], but it is being cultivated more and more for its antimalarial purposes. Although it was confirmed that artemisinin and artesunate in sweet wormroot (*A. annua*) have antiviral activities [52] and application of *A. annua* prevents replication of the coronavirus in in-vitro cells, its effectiveness against COVID-19 in humans was not tested [53].

Kapepula et al. [54] report exaggerated claims of efficacies of *Artemisia* spp. among other herbal methods against COVID-19 in low-income countries. Furthermore, in the search for effective drugs, the two antimalarial drugs chloroquine (CQ) and hydro-chloroquine (HCQ) were the centre of public attention as they were promoted as being effective against COVID-19 by several authorities [55–57]. Ramesh et al. [58] report that there is no benefit of using chloroquine when treating COVID-19 patients. Gasmi et al. [59] state that no significant results are available indicating that chloroquine reduces mortality in COVID-19 patients. However, not clinically confirmed, the media attention of *A. annua* and CQ and HCQ reinforced the participants perceived link between COVID-19 and malaria. As everyone in DR Congo is used to treating malaria in their own way, this alleged link seems to have opened up a range of possibilities and possible remedies to treat COVID-19 curatively by reshaping and adapting traditional knowledge.

Furthermore, a review of the ethnobotanical literature confirms the use against malaria in Sub-Sahara Africa of all other plants cited by our respondents, except for chives (*Allium schoenoprasum* L.) [31, 60–65]. The use of ‘confo’, a mix of essential oils sold online as mosquito repellent and respiratory tract therapeutics was used to cure COVID-19, can be derived from the eucalyptus-like odour of the essential oil mixture, as eucalyptus was used by participants against (symptoms of) COVID-19.

TCAM use in the light of COVID-19 among migrant groups thus also illustrates the resilience of ethnopharmacological knowledge systems, as the strong ability to adapt and transform their TCAM use in the face of a completely new disease incorporates capacity to learn and develop alternative medicinal systems by combining knowledge, experience with new information in response to sudden change [66–68].

Social media and online communication platforms played an essential role in the dissemination of (both official (governmental) and unofficial (non-governmental)) information in general and in the information-seeking strategy of the participants in our study. Information and communication technologies cause virtual transnational social spaces to emerge within transnational social spaces, creating a co-presence of migrants and their acquaintances in multiple worlds at once [69]. Villa-Torres et al. [70] indicate the importance of (virtual) transnational social ties in the health practices of migrant populations, as it can provide social support for people with a migratory background and can serve as a platform for resistance when people feel disconnected to or marginalized by their new country of residence [71]. Through a revolution in communication technology and mass media, there is a time and space compression regarding ethnobotanical practices and knowledge sharing [72].

Moreover, the use of TCAM was closely interlinked with a low institutional and biomedical trust displayed in interviewees’ narratives. Participants especially showed a continuum of scepticism towards the COVID-19 vaccination campaign. Narratives on perceptions towards the COVID-19 vaccine ranged from knowing a better solution (TCAM) over doubts on the reasons and dangers behind the quick development to mistrust in the governmental intentions with the vaccination campaign. It became clear that among participants, biomedical mistrust partly lay at the base of scepticism towards the vaccination campaign. Historical vaccination (and other clinical) trials in Africa, and with people from African descent like the Tuskegee experiment [73], have had disastrous outcomes [74]. These experiments, in which people from African descent were recruited as subjects used for unsafe and cheap clinical trials without informed consent, are clearly a form of medical colonialism [75]. Stories and ideas spread and shared within virtual transnational social spaces were adopted in Belgium. However, these stories cause mistrust in medical care among the involved communities. Brandon et al. [76] state that medical care mistrust among African Americans stems most likely from general mistrust of societal institutions. Low levels of institutional trust leading to scepticism towards the vaccine and the government dealing with the corona pandemic were confirmed through these virtual transnational spaces. In this context, Europe’s colonial history, and in particular the very troubled colonial history of Belgium in DR Congo, cannot be denied. It is believed that the racial discrimination by the Belgian government during the colonial period caused a low institutional trust among Congolese then and even still now [77, 78]. Social and institutional (mis)trust are transferred intergenerationally among children of immigrants [9, 79]. This means that for older immigrants of Congolese descent, their institutional trust might carry traces from their parents’ trust during colonial times.

We thus would like to add to Vanthomme et al. [2] that the sensitivity of sub-Saharan African migrant groups in Belgium to health-threatening situations, like the corona pandemic, may be exacerbated by different levels of mistrust in societal institutions resulting from both historical and present interactions between these groups and other social/ethnic groups. As the effectiveness of measures to reduce virus spread depends mainly on the level of social and (bio-)medical trust and collective societal action supported by integration among key groups such as citizens, institutions, information providers and elected officials [80], quantitative research investigating the impact of low institutional trust of migrant groups on their health and health care-seeking behaviour in Western settings is an absolute must.

Based on our findings, we propose the following policy recommendations. General practitioners and, by extension, health professionals working with migrant groups should be aware of patients' personal preferences and practices regarding health care in their anamneses to develop a culturally sensitive approach to health services. Governmental campaigns such as the COVID-19 vaccination campaign, which involve migrant groups, should adopt a culturally sensitive approach, working with key figures and/or confidants from within the community. Finally, more budget should be made available for research on TCAM active compounds and their effects when used in combination with other (biomedical) health care practices.

## Conclusion

In the context of the COVID-19 pandemic, participants from Congolese (DR Congo) descent in our study retreated to, reshaped and adapted traditional and culturally bound knowledge in order to deal with all aspects of the crisis. Consequently, TCAM use to prevent, cure and fight symptoms of COVID-19 prevailed among the Congolese community in Belgium. *Artemisia annua* L. was the most frequently used herbal medicine. A perceived link between malaria and COVID-19 was made based on their symptomatic similarities and the promotion of both herbal and biomedical antimalarial medicine. Institutional (and biomedical) distrust was loudly voiced and mixed with mistrust in institutionally spread messages on the COVID-19 pandemic, leading to back-folding to participants’ own identity through (digital) transnational networks, and vaccination hesitancy. As this is an exploratory study, more in-depth—digital—ethnobotanical research is needed to uncover (the mechanisms behind) medicinal plant use among the Congolese community in Belgium, and among migrant communities in Western urban settings in general, in the context of new health-threatening situations, such as the COVID-19 pandemic.

## Data Availability

The data sets used and/or analysed during the current study are available from the corresponding author on reasonable request.

## Abbreviations

COVID-19: Coronavirus disease 2019
DR Congo: Democratic Republic of Congo
TCAM: Traditional, complementary and alternative medicine
